# Ability of HIV-1 Nef to downregulate CD4 and HLA class I differs among viral subtypes

**DOI:** 10.1186/1742-4690-10-100

**Published:** 2013-09-16

**Authors:** Jaclyn K Mann, Helen Byakwaga, Xiaomei T Kuang, Anh Q Le, Chanson J Brumme, Philip Mwimanzi, Saleha Omarjee, Eric Martin, Guinevere Q Lee, Bemuluyigza Baraki, Ryan Danroth, Rosemary McCloskey, Conrad Muzoora, David R Bangsberg, Peter W Hunt, Philip JR Goulder, Bruce D Walker, P Richard Harrigan, Jeff N Martin, Thumbi Ndung’u, Mark A Brockman, Zabrina L Brumme

**Affiliations:** 1 HIV Pathogenesis Programme, University of KwaZulu-Natal, Durban, South Africa; 2 KwaZulu-Natal Research Institute for Tuberculosis and HIV, University of KwaZulu-Natal, Durban, South Africa; 3 Mbarara University of Science and Technology, Mbarara, Uganda; 4 University of California San Francisco, San Francisco, CA, USA; 5 Department of Molecular Biology and Biochemistry, Simon Fraser University, Burnaby, BC, Canada; 6 Faculty of Health Sciences, Simon Fraser University, Burnaby, BC, Canada; 7 British Columbia Centre for Excellence in HIV/AIDS, Vancouver, BC, Canada; 8 Massachusetts General Hospital and Harvard University, Boston, MA, USA; 9 Department of Paediatrics, University of Oxford, OX1 3SY, United Kingdom; 10 Ragon Institute of MGH, MIT and Harvard University, Cambridge, MA, USA; 11 Howard Hughes Medical Research Institute, Chevy Chase, MD, USA; 12 Max Planck Institute for Infection Biology, Chariteplatz, Berlin D-10117, Germany

**Keywords:** HIV/AIDS, Nef, Viral diversity, Pathogenesis, CD4, HLA class I

## Abstract

**Background:**

The highly genetically diverse HIV-1 group M subtypes may differ in their biological properties. Nef is an important mediator of viral pathogenicity; however, to date, a comprehensive inter-subtype comparison of Nef *in vitro* function has not been undertaken. Here, we investigate two of Nef’s most well-characterized activities, CD4 and HLA class I downregulation, for clones obtained from 360 chronic patients infected with HIV-1 subtypes A, B, C or D.

**Results:**

Single HIV-1 plasma RNA Nef clones were obtained from N=360 antiretroviral-naïve, chronically infected patients from Africa and North America: 96 (subtype A), 93 (B), 85 (C), and 86 (D). Nef clones were expressed by transfection in an immortalized CD4+ T-cell line. CD4 and HLA class I surface levels were assessed by flow cytometry. Nef expression was verified by Western blot. Subset analyses and multivariable linear regression were used to adjust for differences in age, sex and clinical parameters between cohorts. Consensus HIV-1 subtype B and C Nef sequences were synthesized and functionally assessed. Exploratory sequence analyses were performed to identify potential genotypic correlates of Nef function. Subtype B Nef clones displayed marginally greater CD4 downregulation activity (p = 0.03) and markedly greater HLA class I downregulation activity (p < 0.0001) than clones from other subtypes. Subtype C Nefs displayed the lowest *in vitro* functionality. Inter-subtype differences in HLA class I downregulation remained statistically significant after controlling for differences in age, sex, and clinical parameters (p < 0.0001). The synthesized consensus subtype B Nef showed higher activities compared to consensus C Nef, which was most pronounced in cells expressing lower protein levels. Nef clones exhibited substantial inter-subtype diversity: cohort consensus residues differed at 25% of codons, while a similar proportion of codons exhibited substantial inter-subtype differences in major variant frequency. These amino acids, along with others identified in intra-subtype analyses, represent candidates for mediating inter-subtype differences in Nef function.

**Conclusions:**

Results support a functional hierarchy of subtype B > A/D > C for Nef-mediated CD4 and HLA class I downregulation. The mechanisms underlying these differences and their relevance to HIV-1 pathogenicity merit further investigation.

## Background

HIV-1 Nef is a 27-35 kDa myristoylated accessory protein that promotes viral infectivity, replication and evasion of host immune responses by manipulating several cellular pathways [1-4]. The most well studied of Nef’s diverse functions include cell-surface downregulation of the HIV-1 receptor CD4 as well as HLA class I (HLA-I) molecules that present viral epitopes to CD8+ T cells. Nef-mediated CD4 downregulation enhances release of fully infectious virions expressing HIV-1 envelope, thereby promoting viral infectivity and replication [5-7]. Downregulation of HLA-A and -B molecules promotes evasion of the host immune response, while selective retention of HLA-C surface expression allows infected cells to avoid recognition by natural killer cells [8].

Nef plays an important role in HIV-1 pathogenesis. Nef deletion dramatically impairs SIV pathogenicity in rhesus macaques [9], and slow or non-progression to symptomatic disease has been observed in humans infected with rare Nef-deleted HIV-1 strains [10,11]. Furthermore, despite the general lack of gross mutational defects in Nef among most HIV-1 elite controllers and long-term non-progressors (LTNP) [12-14], one or more Nef functions may be reduced in these individuals [13,15,16], suggesting that more subtle variations in Nef activity may modulate HIV-1 disease outcomes.

HIV-1 Nef sequences are highly diverse, with inter-subtype nucleic acid variation ranging from 14.4% to 23.8% [17]. As such, Nef functions could vary among viral subtypes. Indeed, differences in CD4 and HLA-I downregulation activity have been demonstrated in single patient-derived subtype B and D Nef clones [18], while a patient-derived subtype C Nef demonstrated slightly reduced ability to downregulate CD4 and HLA-I compared to single subtype B, BF, and F Nef clones derived from reference strains [19]. Defects in Nef-mediated up-regulation of the HLA-II-associated invariant chain (Ii) CD74 were also reported for the subtype C and F Nef molecules in the same study [19]. Furthermore, a recent study demonstrated HLA-I downregulation defects in two subtype C and one group O Nef protein, and more efficient CXCR4 downregulation in HIV-2 compared to HIV-1 Nef proteins [20]. While these studies suggest inter-subtype differences in Nef function, assessment of single or few sequences may not be representative of the subtype as a whole. Large-scale functional comparisons of patient-derived Nef sequences from multiple HIV-1 subtypes are therefore required to address this issue.

HIV-1 subtypes may differ in their biological properties, which can in turn impact their transmissibility or pathogenic potential [21-23]. Understanding the basis of such differences could be relevant to the development of prevention or therapeutic strategies [22]. For example, subtype C isolates appear to have lower replication capacity but equivalent, or increased, transmissibility compared to those from other M group subtypes [21,22,24]. These somewhat paradoxical observations have been hypothesized to at least partially explain the global predominance of subtype C [21]. Inter-subtype functional comparisons have also demonstrated differences in the activities of envelope gp120 [25], protease [26], reverse transcriptase [27], vif [28], and long terminal repeat regions [29], but to date no studies have comprehensively evaluated Nef function in circulating HIV-1 sequences of different subtypes.

Given the important role of Nef in HIV-1 pathogenesis, we investigated inter-subtype differences in two of Nef’s most well-characterized functions, CD4 and HLA-I downregulation, using 360 plasma HIV-1 RNA-derived subtype A, B, C and D Nef sequences derived from unique untreated, chronically infected patients. Overall, we observed a hierarchy of subtype B > A/D > C for both Nef-mediated CD4 and HLA-I downregulation function.

## Results

### Selection and functional assessment of Nef clones

**Figure 1:**
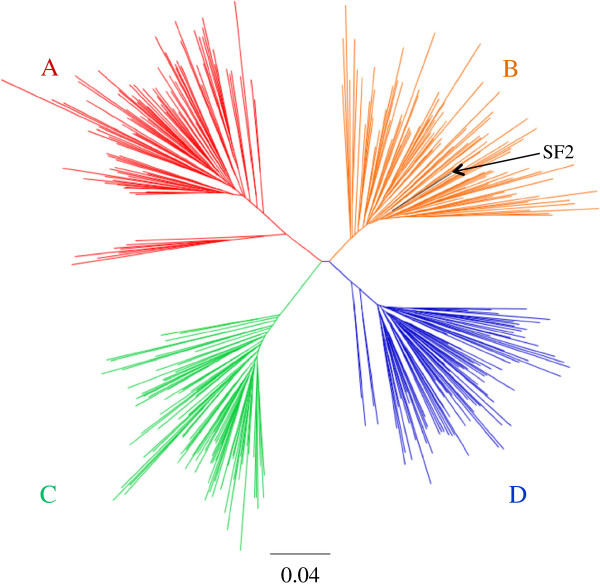
**Maximum likelihood phylogenetic tree of patient-derived HIV-1 Nef clones.** HIV-1 subtype **A** (red), **B** (orange), **C** (green), and **D** (blue) Nef sequences form distinct clusters within the tree. The control SF2 Nef sequence (subtype B) is shown in black.

**Figure 2:**
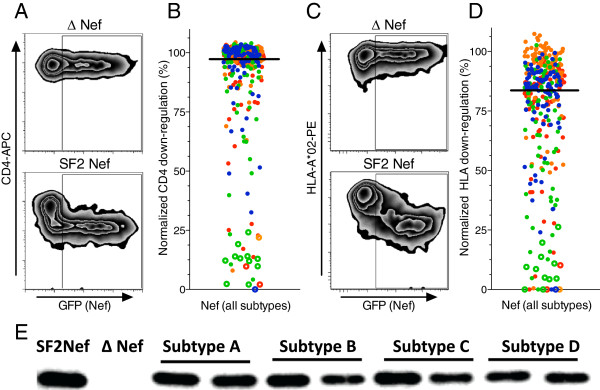
**Function and Nef expression of patient-derived HIV-1 Nef clones. ***Panel ****A****:* Representative flow cytometry plots depict expression of surface CD4 (y-axis) and green fluorescent protein (GFP, x-axis), a marker of Nef-transfected cells, in control experiments. Cells transfected with empty plasmid (∆ Nef, negative control) and cells transfected with wild-type Nef plasmid (SF2 Nef, positive control) are shown. *Panel ****B***: CD4 downregulation activities of subtype **A** (red), **B** (orange), **C** (green) and **D** (blue) Nef clones are shown. Open circles identify Nef clones that displayed poor protein expression by Western blot. The solid black bar represents the median downregulation function, normalized to SF2 Nef. *Panel ****C***: Representative flow cytometry plots depict expression of surface HLA-A*02 (y-axis) and GFP (x-axis) in control experiments, as described in panel **A**. *Panel ****D***: HLA-I downregulation activities of subtype **A** (red), **B** (orange), **C** (green), and **D** (blue) Nef clones are shown. Open circles identify Nef clones that displayed poor expression by Western blot. The solid black bar represents the median downregulation function, normalized to SF2 Nef. *Panel ****E***: Detection of Nef clones by Western blot using rabbit anti-Nef serum. Cells transfected with control SF2 Nef, ∆Nef, and two representative patient-derived Nef clones from each subtype are shown.

A single plasma HIV-1 *nef* sequence from each of 96 (subtype A), 93 (B), 85 (C), and 86 (D) individuals, whose characteristics are summarized in Table 1, was cloned into an expression plasmid featuring independent promoters for *nef* and green fluorescent protein (GFP). Nef clones clustered closely with their respective bulk plasma HIV RNA sequences in a phylogenetic tree (not shown), were free of gross genetic defects and were clearly classifiable as subtype A, B, C or D by phylogenetic analysis (Figure 1). Following transient transfection of each Nef expression plasmid into a CEM-derived T cell line, Nef-mediated downregulation of CD4 and HLA-A*02 was assessed by flow cytometry and normalized to that of the control subtype B strain Nef-SF2 (Figure 2). Concordance between replicate measurements was excellent for CD4 and HLA-I downregulation assays (Spearman’s, r = 0.97 and p < 0.0001 for both; not shown). Overall, the CD4 downregulation function of patient-derived Nef clones ranged from 0% to 104% relative to the activity of a positive control Nef-SF2 (median [inter-quartile range; IQR] 99% [94-101]) (Figure 2A and B), while HLA-I downregulation function ranged from 0% to 107% relative to Nef-SF2 (median [IQR] 86% [72-93]) (Figure 2C and D).

**Table 1:** Characteristics of study subjects infected with HIV-1 subtypes A, B, C and D

	**Subtype A**	**Subtype B**	**Subtype C**	**Subtype D**	**p-value**^**b**^
	**N = 96**	**N = 93**	**N = 85**	**N = 86**	
Sex (% female)	61	7.5	80	80	<0.0001
Age (years)^a^	35 (30–40)	37 (32–44)	31 (27–37)	33 (29–38)	<0.0001
Plasma HIV-1 RNA (log_10_copies/ml)^a^	4.95 (4.55–5.59)	5.28 (5.08–5.66)	4.65 (4.26–5.24)	4.88 (4.45–5.29)	<0.0001
CD4 count (cells/mm^3^)^a^	154 (88–198)	200 (80–360)	369 (243–466)	128 (73–216)	<0.0001

^a^Medians with inter-quartile ranges in brackets are shown.

^b^p-values calculated using the Kruskal-Wallis Test.

### Verification of Nef protein expression by Western blot

Despite harboring an intact reading frame and no evidence of gross sequence defects, 22 of 360 (6.1%) Nef clones (4% subtype A, 2% subtype B, 16% subtype C and 2% subtype D) displayed poor relative function (<35% that of control Nef-SF2) for both CD4 and HLA-I downregulation (Figure 2B and D). To test whether poor Nef function could be attributable to impaired protein expression or stability, Western blot analysis was performed on all 22 poorly functional clones as well as a random selection of 60 clones with CD4 and HLA-I downregulation activities above this threshold (Figure 2E). Of the 22 poorly functional clones, 15 (68.2%) displayed weak or no detection of Nef protein by Western blot (Figure 2B and D), whereas of the 60 randomly selected functional clones, all but 3 were readily detected. Furthermore, excluding the 15 poorly functional and poorly expressed clones, no significant differences in Western blot band intensity were observed among HIV-1 subtypes (ANOVA, p = 0.83; not shown). Although it is possible that the 15 poorly functional and poorly detected clones represent Nef sequences that were defective *in vivo*, we cannot rule out *in vitro* expression or stability defects resulting from RT-PCR or cloning artifacts. Thus, to be conservative, all 15 poorly functional clones that could not be validated by Western blot were excluded from subsequent analyses.

### Inter-subtype comparison of Nef-mediated CD4 and HLA-I downregulation

**Figure 3:**
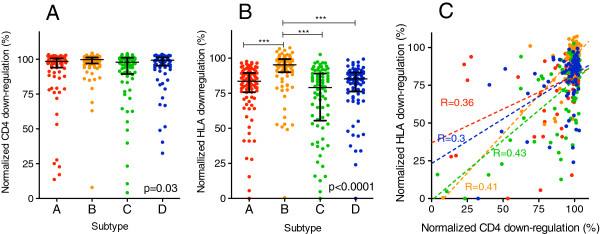
**Inter-subtype comparison of Nef-mediated CD4 and HLA-I downregulation capacities. ***Panels ****A ****and ****B****:* CD4 **(A)** and HLA-A*02 **(B)** downregulation activities of subtype **A** (red), **B** (orange), **C** (green) and **D** (blue) patient-derived Nef clones are shown. Bars represent the median and whiskers represent the inter-quartile range for each group. Kruskal-Wallis with Dunn’s multiple comparisons post-hoc tests were used to compare Nef functions between subtypes. The Kruskal-Wallis p-value is shown. In addition, significant differences between individual groups are indicated by asterisks above the bar indicating the two groups compared. The number of asterisks denotes the level of significance, namely, p < 0.05 (*), p < 0.01 (**) and p < 0.001 (***). *Panel ****C****:* CD4 and HLA-I downregulation functions within each subtype displayed significant positive relationships (Spearman’s, all p < 0.01).

CD4 downregulation activities of HIV-1 subtype B Nef clones were marginally yet significantly higher than the other subtypes examined (median [IQR] subtype A, 98% [94-101]; B, 100% [97-101]; C, 98% [90-101]; D, 99% [96-101]) (Kruskal-Wallis, p = 0.03; Figure 3A). In contrast, Nef-mediated HLA-I downregulation activity differed markedly among subtypes (median [IQR] subtype A, 84% [76-89]; B, 95% [90-99]; C, 79% [57-89]; D, 85% [77-89]) (Kruskal-Wallis, p < 0.0001; Figure 3B). Subtype B Nef clones displayed significantly greater HLA-A*02 downregulation capacities compared to those of all other subtypes tested (Dunn’s multiple comparisons test, all p < 0.001; Figure 3B). We observed a modest association between Nef-mediated CD4 and HLA-I downregulation activities for patient-derived Nef clones within each subtype (Spearman’s, r ≥ 0.3 and p < 0.01; Figure 3C).

### Nef-mediated HLA-A*02 versus HLA-B*07 downregulation

**Figure 4:**
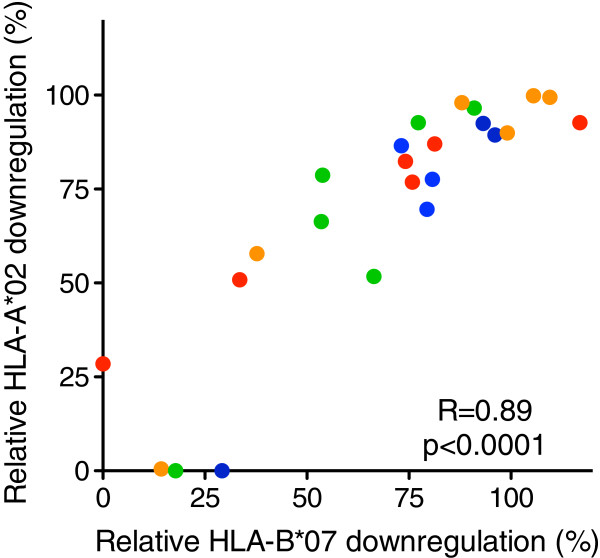
Comparison of Nef-mediated HLA-A*02 and HLA-B*07 downregulation capacities. A strong positive correlation (Spearman’s, r = 0.89 and p < 0.0001) between Nef-mediated HLA-A*02 and HLA-B*07 downregulation capacities of 24 patient-derived Nef clones of subtypes A (red), B (orange), C (green) and D (blue) is shown.

Nef-mediated HLA-I downregulation occurs through a sequence shared by the cytoplasmic tails of HLA-A and HLA-B molecules [3,30], however a recent study suggests that HLA-B molecules are downregulated less efficiently than HLA-A molecules [31]. Therefore, we also assessed the ability of Nef to downregulate HLA-B molecules for a subset of 24 clones spanning all subtypes, using a CEM T-cell line stably expressing HLA-B*07. The correlation between Nef’s ability to downregulate A*02 and B*07 was robust (Spearman’s, r = 0.89 and p < 0.0001; Figure 4), indicating that HLA-A*02 downregulation measurements are generally representative of Nef’s ability to downregulate both HLA-A and -B.

### Addressing demographic and clinical characteristics as potential confounders

Nef function might differ according to HIV-1 disease stage [32,33] and clinical status [13,15,16]. Furthermore, substantial demographic differences are observed in HIV-infected populations globally (Table 1). Although no significant correlations were observed between Nef function and patient viral load or CD4 count in any individual subtype (all p > 0.05; not shown), we nevertheless wanted to control for differences in age, sex, and clinical parameters among cohorts. We did so using two approaches. First, we restricted our analysis to a subset of patients who were matched for viral load and CD4 cell counts. Second, we conducted a multivariable analysis adjusting for patient demographic and clinical characteristics directly.

**Figure 5:**
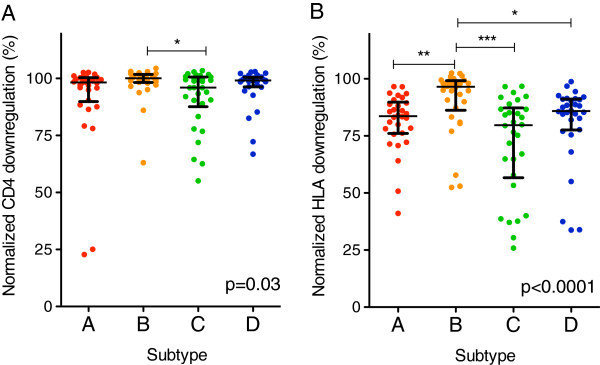
**Inter-subtype comparison of Nef-mediated CD4 and HLA-I downregulation capacities in a subset of individuals matched for plasma viral load and CD4 count. ***Panels ****A ****and ****B****:* CD4 **(A)** and HLA-A*02 **(B)** downregulation activities of subtype **A** (red), **B** (orange), **C** (green) and **D** (blue) patient-derived Nef clones from selected individuals matched for patient plasma viral loads and CD4 counts are shown (n = 30 per subtype). Bars and whiskers represent the median and inter-quartile ranges, respectively. Kruskal-Wallis with Dunn’s post-hoc tests were used to compare Nef functions between subtypes. The Kruskal-Wallis p-value is shown and significant differences between individual subtypes are indicated by asterisks; p < 0.05 (*), p < 0.01 (**) and p < 0.001 (***).

Firstly, we identified 30 patients per subtype with comparable plasma viral loads (median [IQR] 5.11 log_10_ copies/ml [4.73-5.50], p = 0.92) and CD4 counts (overall median [IQR] 199 cells/mm^3^ [89-286], p = 0.7). Despite reduced statistical power, Nef function in this clinically-matched subset was consistent with our original observations (Kruskal-Wallis, p = 0.03 and p < 0.0001 for inter-subtype differences in CD4 and HLA-I downregulation activities, respectively; Figure 5). Furthermore, the relative hierarchy of Nef function and the magnitude of inter-subtype differences also remained consistent: subtype B Nef clones from clinically-matched participants exhibited modest yet significantly higher CD4 downregulation activities than those from subtype C (median [IQR] subtype A, 98% [92-100]; B, 100% [98-102]; C, 96% [89-100]; D, 99% [97-100]) (Dunn’s multiple comparisons tests, p < 0.05; Figure 5A). Subtype B Nef clones also displayed significantly higher HLA-I downregulation activities than those from subtypes A, C and D (median [IQR] subtype A, 84% [77-89]; B, 97% [88-99]; C, 80% [60-87]; D, 86% [79-91]) (Dunn’s multiple comparisons tests, p < 0.05; Figure 5B).

Secondly, using the entire dataset, we performed univariate and multivariable linear regression to investigate the relationship between Nef function and HIV-1 subtype, log_10_ plasma viral load, logCD4 count, sex and age (Table 2). In the multivariable analyses, log_10_ plasma viral load remained significantly associated with Nef-mediated CD4 downregulation (with a 3.3% increase in CD4 downregulation activity per log_10_ plasma viral load increase, p = 0.02), while a trend remained for subtype A and C Nef clones having on average 4.8% (p = 0.06) and 5.0% (p = 0.07) lower CD4 downregulation activity, respectively, than those from subtype B after adjusting for log_10_ plasma viral load (Table 2). In the multivariable analysis of Nef-mediated HLA-I downregulation, HIV-1 subtype emerged as the single significant predictor of function, with subtypes A, C and D Nef clones exhibiting adjusted averages of 16%, 24% and 16% lower function, respectively, compared to those from subtype B (p < 0.0001) (Table 2).

**Table 2:** Linear regression models investigating the relationship between Nef function and socio-demographic characteristics, clinical parameters and HIV-1 subtype

**Variable**		**Univariate (CD4)**		**Multivariable (CD4)**^**a**^		**Univariate (HLA-I)**		**Multivariable (HLA-I)**^**b**^	
		**Estimate**	**p-value**	**Estimate**	**p-value**	**Estimate**	**p-value**	**Estimate**	**p-value**
Subtype	A	-0.060	0.02	-0.048	0.06	-0.14	<0.0001	-0.16	<0.0001
	B	0	N/A	0	N/A	0	N/A	0	N/A
	C	-0.072	0.007	-0.051	0.07	-0.21	<0.0001	-0.24	<0.0001
	D	-0.028	0.3	-0.012	0.7	-0.13	<0.0001	-0.16	<0.0001
Log viral load		0.040	0.002	0.033	0.02	0.050	0.003	-	-
Log CD4		-0.009	0.7	-	-	-0.013	0.6	-	-
Male		0.026	0.2	-	-	0.057	0.02	-0.06	0.06
Age		-0.0006	0.6	-	-	0.000001	0.94	-	-

β Estimates for log viral load and log CD4 count are expressed per log_10_ increment. Age is expressed per year increment. For sex, female is the reference group. For HIV-1 subtype, B is the reference group.

^a^Multivariable model for CD4 downregulation: multiple r^2^ = 0.045, p = 0.006.

^b^Multivariable model for HLA-I downregulation: multiple r^2^ = 0.14, p < 0.0001.

### Inter-subtype differences in Nef function observed using subtype consensus sequences

**Figure 6:**
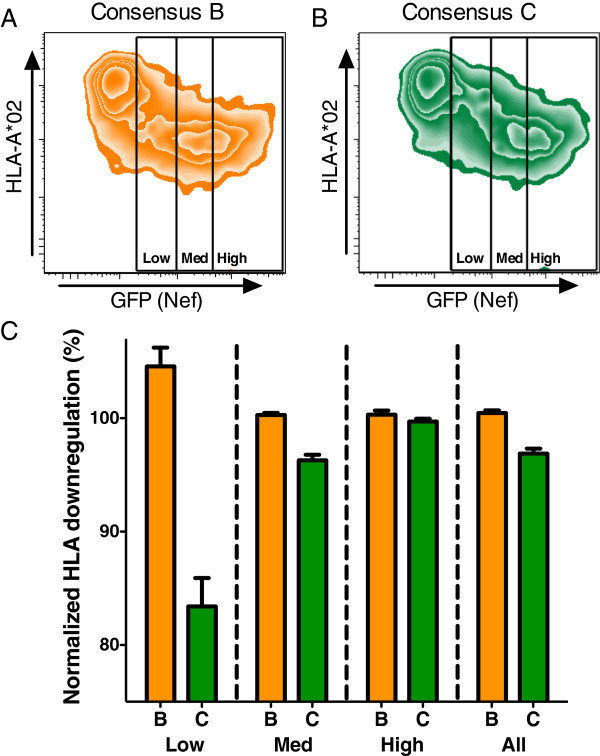
**Analysis of Nef HLA-I downregulation capacity for LANL 2004 consensus B and C Nef sequences. ** Consensus B and C Nef sequences were obtained from the Los Alamos National Laboratory HIV Sequence database (2004 update), synthesized and tested. *Panels ****A ****and ****B****:* Representative flow cytometry plots depict expression of surface HLA-A*02 (y-axis) and green fluorescent protein (GFP, x-axis), a marker of Nef-transfected cells. Representative flow cytometry plots for cells transfected with consensus B Nef plasmid **(A)** or consensus C Nef plasmid **(B)** are shown. HLA-I downregulation activity was assessed in tertiles of cells expressing low, medium (med), or high amounts of Nef, as indicated by the level of GFP co-expressed by the dual promoter plasmid. *Panel ****C***: Results from ten replicate assays are shown, indicating a reduced ability of consensus C Nef to downregulate HLA-I, particularly in cells expressing the lowest amount of Nef protein. For comparison, the outcome when all cells are gated together is also presented. Bars and whiskers represent the mean and standard error of the mean for each group, respectively.

To further investigate inherent differences in Nef function among HIV-1 group M subtypes, we synthesized, cloned and assessed *in vitro* CD4 and HLA-I downregulation activities of the consensus Nef sequences for subtype B (which was anticipated to display highest function) and subtype C (which was anticipated to display the lowest function) (2004 consenses, available at http://www.hiv.lanl.gov/content/sequence/NEWALIGN/align.html). Differences in Nef function were noted between subtype consenses, which was most apparent for HLA-I downregulation and appeared to be dependent on Nef protein expression levels (Figure 6A, 6B). Whereas consensus B Nef efficiently downregulated HLA-I at low protein concentrations (Figure 6A), greater doses of consensus C Nef were required to downregulate HLA-I to the same extent (Figure 6B). Indeed, when HLA-I downregulation data were analyzed according to tertiles of protein expression, consensus C Nef displayed 21.2% lower activity than consensus B Nef in cells expressing low amounts of protein, 4.0% lower activity in cells expressing moderate levels of protein, and equivalent (0.6% lower) activity in cells expressing the highest amounts of Nef protein, yielding an overall difference of 3.6% (Figure 6C). A similar, although less marked dose-dependent relationship was observed for Nef-mediated CD4 downregulation, with consensus C Nef displaying 6.1% lower activity than consensus B Nef in cells expressing low amounts of protein, and 1.5% lower activity overall (not shown).

### Genotypic correlates of differential Nef function

**Figure 7:**
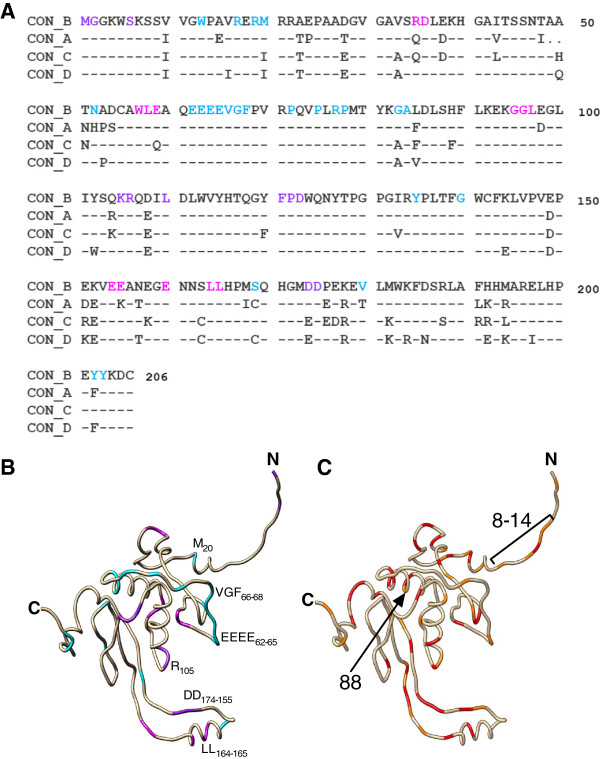
**Inter-subtype comparison of Nef sequences in the context of known Nef functional motifs. ***Panel ****A****:* Consensus **A**, **B**, **C** and **D** Nef sequences were determined using patient-derived clones from this study and are shown aligned to HXB2. Amino acid residues/motifs previously associated with Nef-mediated modulation of CD4 downregulation (magenta), HLA-I downregulation (cyan) or both (purple) are indicated. Insertions were observed at codons 23, 24, 25, 63, 64, and 65 in greater than 10% of clones for at least one subtype (indicated by asterisks). *Panel ****B****:* The position of residues/motifs associated with Nef CD4 downregulation (magenta), HLA-I downregulation (cyan), or both functions (purple) are highlighted on a structure model of the Nef protein (composite crystal structure kindly provided by Art F. Y. Poon, [44]). *Panel ****C***: The position of amino acids whose sequence varied between consensus B and C Nef sequences from this study are shown using a structure model of Nef. Differences between consensus sequences (32 residues) are highlighted in red and majority amino acids whose frequency varied by greater than 15% between **B** and **C** (additional 29 residues) are indicated in orange.

As expected [17,34], Nef clones exhibited substantial inter- and intra-subtype diversity (HXB2-aligned clonal Nef amino acid sequences provided in Additional file 1: Table S1). Cohort consensus Nef amino acid sequences for subtypes A-D differed at 52 of 206 codons (25%) (Figure 7A), of which 10 differences were positioned at residues or within motifs reported to be involved in modulating CD4 or HLA-I downregulation [1,2,20,35-43] (Figure 7A and 7B). A comparison of amino acid frequencies between subtype B and C sequences identified 32 residues (15.5%) that differed in their consensus amino acid, and a further 29 residues (14.1%) that differed by at least 15% in their major amino acid frequency (Figure 7C). It is likely that genotypic determinants of inter-subtype Nef functional differences include amino acids at one or more of these sites.

Next, we examined genotypic determinants of Nef function within subtypes and then assessed whether these could also shed light on observed functional differences between subtypes. Firstly, Nef amino acid length (median 206, 207, 207, 208 residues in subtypes A, B, C and D, respectively) did not correlate with CD4 or HLA-I downregulation activity for any of the subtypes (Spearman’s, r ≤ 0.17 and p ≥ 0.1; not shown). Secondly, intra-subtype analyses identified 33 amino acids occurring at 22 unique Nef codons that were associated with differential HLA-I downregulation activity in at least one subtype (p < 0.05 and q ≤ 0.4; Table 3). However, using this approach, no Nef residues were identified as being associated (at q < 0.4) with CD4 downregulation function in any subtype. Thirdly, as we were particularly interested in sequence correlates for the least functional Nef clones, we compared amino acid frequencies in subtype C Nef sequences from the highest vs. lowest functional quartiles for CD4 downregulation. Of interest, the lowest-function subtype C Nef clones were enriched for non-consensus 88G while the highest-function clones were 100% conserved for consensus 88S (Bonferroni-corrected p = 0.02). No additional amino acids were identified following Bonferroni correction in a similar analysis undertaken for HLA-I downregulation; however, we noted that subtype C Nef clones with the lowest HLA-I downregulation function displayed higher sequence conservation at Nef codons 8-12 (Student’s T test, p = 0.0006). Finally, we assessed whether Nef residues associated with intra-subtype differences in function also varied in frequency among subtypes [45] (Table 4). Of the 36 Nef residues associated with intra-subtype function, 33 also displayed significantly different inter-subtype frequencies. Of interest, amino acid frequencies in the region spanning codons 8-14 and at codon 88 (all p < 0.05 and q < 0.05; Table 4 and Figure 7C) were coincident with functional differences observed between subtypes.

**Table 3:** Amino acids associated with intra-subtype Nef HLA-I downregulation function

**Subtype**	**Codon**^**a**^	**AA**^**b**^	**Cons.**^**c**^	**Function**^**d**^		**No. of samples**^**e**^		**p-value**	**q-value**
				**+ AA**	**- AA**	**+ AA**	**-AA**		
A	3	G	G	82.3	87.1	71	23	0.007	0.4
	9	R	S	94.5	83.1	5	84	0.02	0.4
	11	V	V	81.5	87.9	74	16	0.007	0.4
	83	G	G	80.6	87.0	63	31	0.01	0.4
	89	F	H	90.8	82.4	15	79	0.01	0.4
	105	R	K	78.4	85.0	22	72	0.02	0.4
	120	Y	Y	82.4	86.7	56	38	0.02	0.4
	168	M	I	87.1	81.9	23	71	0.02	0.4
	191	L	L	81.0	87.1	57	37	0.005	0.4
	196	Q	R	80.2	86.2	34	60	0.008	0.4
B	8	R	S	98.2	94.6	28	56	0.006	0.2
	8	S	S	94.3	98.0	38	46	0.0009	0.2
	14	P	P	94.4	97.7	58	34	0.01	0.2
	28	A	D	101.5	95.0	7	85	0.004	0.2
	65	D	E	89.7	96.4	8	84	0.02	0.3
	65	E	E	96.4	87.3	83	9	0.009	0.2
	89	F	H	63.7	95.6	5	87	0.03	0.4
	89	H	H	95.7	60.8	86	6	0.007	0.2
	98	D	E	99.7	95.1	6	86	0.02	0.3
	98	E	E	95.0	100.6	85	7	0.007	0.2
	105	Q	K	91.0	96.5	18	74	0.03	0.4
	153	I	V	90.5	96.7	16	75	0.007	0.2
	153	V	V	96.7	90.0	74	17	0.003	0.2
	198	K	L	98.8	94.7	19	73	0.01	0.2
	202	F	Y	99.4	95.0	9	83	0.01	0.2
	202	Y	Y	95.0	99.4	83	9	0.01	0.2
C	9	S	S	78.1	96.6	66	5	0.01	0.3
	40	H	H	84.0	72.3	38	36	0.005	0.3
	40	Y	H	72.3	84.0	34	40	0.003	0.3
	102	H	Y	56.2	81.8	12	62	0.02	0.4
	105	K	K	83.5	71.1	53	21	0.005	0.3
	105	R	K	57.8	82.5	15	59	0.01	0.3
	108	D	E	71.1	83.7	37	37	0.01	0.3
	108	E	E	83.7	71.1	37	37	0.01	0.3
	182	Q	K	49.6	80.7	8	66	0.01	0.3

^a^Numbered according to HXB2.

^b^ Amino acid variants associated with differences in HLA-I downregulation function. Only common amino acid variants (present at n ≥ 5 in each subtype) were investigated.

^c^Consensus amino acid for the codon and subtype indicated.

^d^Median HLA-I downregulation function (expressed as a percentage relative to SF2) of Nef sequences with (+AA) and without (-AA) the amino acid variant.

^e^Number of sequences with (+AA) and without (-AA) the amino acid variant. Amino acid totals vary, as gaps in the alignment are considered missing data.

A similar intra-subtype analysis was performed to identify Nef amino acids associated with CD4 downregulation function; no associations q < 0.4 were identified using this approach.

**Table 4:** Frequencies of amino acids associated with altered intra-subtype Nef function

**Function**	**Codon**^**a**^	**AA**	**Subtype**^**b**^	**+/-**^**c**^	**% Frequency**				**p-value**	**q-value**
					**A**	**B**	**C**	**D**		
CD4	88	G	C	-	10	2	20	2	<0.0001	<0.0001
	88	S	C	+	90	97	80	98	0.0001	0.0001
HLA-I	3	G	A	-	76	95	66	81	<0.0001	<0.0001
	8-12	SSIVG	C	-	82	48^d^	81	88	<0.0001	<0.0001
	8	R	B	+	10	30	7	12	<0.0001	<0.0001
	8	S	B	-	80	41	81	81	<0.0001	<0.0001
	9	R	A	+	5	9	4	0	0.05	0.06
	9	S	C	-	87	70	89	88	0.0008	0.001
	11	V	A	-	79	34	74	88	<0.0001	<0.0001
	14	P	B	-	89	63	93	94	<0.0001	<0.0001
	28	A	B	+	14	8	1	4	0.007	0.008
	40	H	C	+	86	79	51	93	<0.0001	<0.0001
	40	Y	C	-	10	14	46	5	<0.0001	<0.0001
	65	D	B	-	11	9	1	8	0.13	0.13
	65	E	B	+	86	90	97	87	0.08	0.09
	83	G	A	-	67	57	45	31	<0.0001	<0.0001
	89	F	A/B	+/-	16	5	97	5	<0.0001	<0.0001
	89	H	B	+	82	93	0	95	<0.0001	<0.0001
	98	D	B	+	85	7	47	31	<0.0001	<0.0001
	98	E	B	-	15	92	53	69	<0.0001	<0.0001
	102	H	C	-	9	25	16	1	<0.0001	<0.0001
	105	K	C	+	73	54	72	78	0.004	0.005
	105	Q	B	-	2	20	8	13	0.001	0.002
	105	R	A/C	-/-	23	26	20	9	0.03	0.04
	108	D	C	-	15	86	50	45	<0.0001	<0.0001
	108	E	C	+	84	13	50	55	<0.0001	<0.0001
	120	Y	A	-	60	93	35	62	<0.0001	<0.0001
	153	I	B	-	1	17	1	9	<0.0001	<0.0001
	153	V	B	+	99	80	99	87	<0.0001	<0.0001
	168	M	A	+	24	61	76	39	<0.0001	<0.0001
	182	Q	C	-	5	28	11	1	<0.0001	<0.0001
	191	L	A	-	61	3	0	1	<0.0001	<0.0001
	196	Q	A	-	36	0	0	0	<0.0001	<0.0001
	198	K	B	+	10	21	35	33	0.0002	0.0002
	202	F	B	+	62	10	23	62	<0.0001	<0.0001
	202	Y	B	-	35	90	69	38	<0.0001	<0.0001

^a^Numbered according to HXB2.

^b^Indicates the subtype in which the amino acid is associated with altered Nef function.

^c^Indicates whether the amino acid is associated with increased (+) or decreased (-) Nef function.

^d^The consensus for subtype B is SSVVG and is 52% conserved.

Given the extensive inter- and intra-subtype sequence differences observed in Nef, and the expectation that complex codon co-varation pathways are possible [46], identification of underlying genotypic determinants of Nef function will require substantial further study. Towards this end, we constructed S9R and S88G mutants in consensus B and C backbones, and assessed their ability to downregulate HLA-I and CD4, respectively. As expected, introduction of non-consensus 9R increased HLA-I downregulation capacity in both B and C backbones, while introduction of non-consensus 88G reduced CD4 downregulation in both B and C backbones; however, the magnitudes of these effects were modest (≤3%) in all cases (data not shown).

## Discussion

This study investigated differences in two of HIV-1 Nef’s most well-characterized functions, CD4 and HLA-I downregulation, using unique plasma RNA-derived Nef clones obtained from 360 untreated individuals chronically infected with HIV-1 subtype A, B, C or D. While inter-subtype differences in Nef-mediated CD4 downregulation activity were relatively modest, those for HLA-I downregulation were substantial. For both activities, a hierarchy of Nef function was observed, with subtype B > A/D > C. These differences remained statistically significant in analyses of a subset of Nef clones from subjects who displayed similar clinical values for plasma viral load and CD4 cell count. In addition, HIV-1 subtype remained the sole significant correlate of Nef-mediated HLA-I downregulation function in multivariable analyses that controlled for differences in age, sex, and clinical characteristics. After controlling for these other factors, we estimate that naturally occurring HIV-1 Nef sequences differ in their ability to downregulate CD4, on average, by up to 5% between subtypes, whereas downregulation of HLA-I differs, on average, by up to 24% between subtypes (Table 2). In addition, a comparison of consensus subtype B and C Nef sequences confirmed greater *in vitro* function for subtype B Nef, which was most pronounced at low Nef protein concentrations. Further supporting these observations, in an independent study of 73 patient-derived clonal Nef sequences from a different cohort of subtype C-infected South African patients, we observed ~5% and ~24% lower median CD4 and HLA-I downregulation activities, respectively, compared to the subtype B control SF2 Nef [47]. Taken together, our results support significant inherent differences in Nef-mediated HLA-I downregulation function, and possibly modest differences in CD4 downregulation, between HIV-1 subtypes.

Nef-mediated CD4 downregulation promotes HIV-1 infectivity by increasing virion incorporation of envelope [5-7], and this activity correlates with Nef’s ability to enhance viral replication capacity [48]. It is tempting to speculate that the modest reduction in CD4 downregulation activity observed for subtype C Nef clones could result in a lower replication capacity for HIV-1 subtype C strains compared to other M group subtypes [21] – perhaps in conjunction with functional differences in envelope gp120 [25] and reverse transcriptase [27], but not other viral regions [27-29]. Indeed, it has been proposed that lower replication capacity but equal (or perhaps enhanced) transmissibility of subtype C isolates favor their ability to spread, and thus contribute to their higher global prevalence [21,24,49]. Although inter-subtype differences in HIV-1 pathogenicity or rate of disease progression remain controversial [21-23,50-54], it is conceivable that the diversity in Nef functions observed in this study could contribute to inherent differences in the intra- and inter-subtype pathogenicity of globally diverse HIV-1 strains.

Consistent with a recent comparison of HIV-1 group M, group O and HIV-2 Nef sequences [20], intra- and inter-subtype dynamic ranges of HIV-1 Nef-mediated HLA-I downregulation activity were broader than those for CD4 downregulation, suggesting that preservation of Nef’s ability to modulate CD4 may be more essential *in vivo*. Indeed, disruption of Nef motifs involved in CD4 downregulation and enhancement of viral infectivity revealed that these Nef functions were critical for viral replication in early SIV infection, and thus that HLA-I downregulation alone was not adequate for SIV virulence [55]. In addition, although Nef sequences with efficient HLA-I downregulation activity were selected during early SIV and HIV infection [33,56], elimination of this function did not significantly impact SIV viral loads [56]. Furthermore, while Nef-mediated CD4 downregulation is typically maintained or enhanced throughout the HIV-1 disease course [33], Nef’s HLA-I downregulation activity appears to be maintained in chronic infection [48], but is reported to diminish after progression to AIDS [33]. Further studies will be required to fully assess the relative contribution of various Nef functions to HIV-1 pathogenesis [1].

Substantial inter-subtype sequence diversity was observed among Nef clones. For example, between subtypes B and C, nearly one-third of Nef codons differed by at least 15% in their major amino acid frequencies. Similarly, of the 45 residues previously reported to play a role in CD4 and HLA-I downregulation, 16 (35.5%) differed by at least 15% in their major amino acid frequencies between at least two subtypes (Figure 7 and Additional file 1: Table S1), yielding potential candidate domains to examine in future studies aimed at elucidating the mechanisms underlying observed inter-subtype functional differences.

While major determinants of Nef-mediated CD4 and HLA-I downregulation functions appear to be genetically separable [1,33,55], our observation of a modest positive correlation between these activities in patient-derived sequences (Figure 3C) suggests that secondary or shared genetic determinants may also affect these protein activities. To this end, we also undertook exploratory analyses to identify residues associated with intra-subtype differences in Nef function. Several Nef residues identified in this way also differed in frequency between subtypes, highlighting potential additional mediators of observed inter-subtype functional differences. In agreement with our previous study of Nef function in HIV-1 subtype B-infected elite controllers [16], global consensus amino acids 8S, 11 V, and 14P in the N-terminal domain (adjacent to Nef’s myristoylation motif [2]) were associated with lower HLA-I downregulation in intra-subtype analyses. These consensus residues, together with consensus 9S (also associated with decreased HLA-I downregulation in the present study, and subsequently validated by site-directed mutagenesis), were considerably less frequent in subtype B Nef clones compared to those from subtypes A, C and D, consistent with the superior HLA-I downregulation activity of subtype B. Moreover, subtype B Nef clones were overall significantly less well conserved at residues 8-12, and conservation in this region was associated with lower HLA-I downregulation activity in subtype C Nef clones. Similarly, 88S correlated with increased CD4 downregulation activity in subtype C in both clinical isolates and site-directed mutagenesis experiments; however, this residue was least frequent in this subtype, which may explain in part the modest reduction in CD4 downregulation activity observed among subtype C Nef clones.

Some limitations of this study require mention. First, a minority (15 of 360, 4.2%) of Nef clones with poor expression and poor function were excluded from analysis because we could not rule out *in vitro* defects in protein expression or stability resulting from PCR or cloning artifacts. Although our overall results are robust to the inclusion or exclusion of these clones (not shown), it is intriguing that a higher proportion of poorly functional Nef clones were isolated from chronic subtype C-infected individuals. Follow-up studies to test this directly are therefore warranted. Second, only two Nef activities (CD4 and HLA-I downregulation) were assessed in this study. Nef is known to perform multiple *in vitro* functions*,* and it remains uncertain which are the most relevant for *in vivo* HIV-1 pathogenesis [1]. In addition, Nef function may vary depending on the infected cell type [16,57], thus additional work will be necessary to determine whether our observations extend to primary cells and cell types other than CD4+ T-lymphocytes. Finally, the potential confounding effects of socio-demographic and clinical differences among HIV-1 infections globally represent major challenges when investigating inter-subtype differences in viral function or pathogenesis. Our analysis of a relatively large number of Nef clones collected from well-characterized cohorts allowed us to control for these confounding factors and to identify HIV-1 subtype as an independent predictor of Nef-mediated HLA-I downregulation function. Nevertheless, our results merit independent confirmation from additional cohorts, geographical regions, and viral subtypes.

## Conclusions

In conclusion, we observed that the *in vitro* CD4 and HLA-I downregulation activities of patient-derived Nef sequences differed among HIV-1 M group subtypes. We observed a hierarchy of Nef function - subtype B > A/D > C – where inter-subtype differences in CD4 downregulation were relatively modest, while differences in HLA-I downregulation were substantial. Results raise the intriguing hypothesis that differences in Nef protein function may contribute to variation in the pathogenicity of HIV-1 subtypes.

## Methods

### Study subjects

Nef sequences were derived from 360 antiretroviral naïve individuals chronically infected with HIV-1 subtypes A (N = 96), B (N = 93), C (N = 85) and D (N = 86) (Table 1). All 96 subtype A, all 86 subtype D, and two subtype C sequences were derived from two cohorts in Uganda: the Adherence Monitoring Uganda (AMU) cohort from Kampala [58] and the Uganda AIDS Rural Treatment Outcomes (UARTO) cohort from Mbarara [59]. A total of 92 subtype B sequences were derived from the HAART Observational Medical Evaluation and Research (HOMER) Cohort from British Columbia, Canada [46,60]. A total of 83 subtype C sequences and one subtype B sequence were derived from the Sinikithemba cohort from Durban, South Africa [61]. Specimens were randomly selected based on the availability of stored plasma HIV-1 RNA or first-round plasma RNA-derived PCR products spanning the *nef* region ([60] and unpublished data). The study was approved by all relevant institutional review boards; all subjects provided written informed consent or specimens were anonymized according to REB-approved procedures.

### Amplification of *nef*

Amplification of plasma HIV-1 *nef* was performed by nested RT-PCR using subtype-specific first-round primers and high-fidelity enzymes. This was followed by second round PCR using a high-fidelity enzyme (Roche Expand HiFi™) and novel primers capable of amplifying subtypes A, B, C, and D that additionally contained AscI (forward) and SacII (reverse) restriction sites for cloning. The second round primer sequences were: Forward 5′-AGAGCACC**GGCGCGCC***TCCACATACCTASAAGAATMAGACARG*-3′ (Asc I site bolded, HXB2 nucleotides 8746-8772 italicized) and Reverse 5′-GCCT**CCGCGG**ATCGAT*CAGGCCACRCCTCCCTGGAAASKCCC*-3′ (SacII site bolded, HXB2 nucleotides 9474-9449 italicized). Amplicons were purified using the QIAQuick PCR purification kit (Qiagen) prior to cloning.

### Cloning and sequencing of *nef*

The pSELECT-GFPzeo expression plasmid (Invivogen), modified by insertion of a linker containing AscI and SacII restriction sites, was digested with AscI and SacII, and purified by electrophoresis and gel extraction (GeneJet gel extraction kit, Thermo Scientific) to isolate the cut plasmid. Each *nef* PCR product was digested with AscI and SacII and ligated into the cut pSELECT-GFP plasmid using 5U T4 ligase per μg DNA (6:1 molar ratio of *nef* insert to vector). The ligation mixture was transformed into OneShot TOP10 competent cells (Invitrogen) according to manufacturer’s instructions, and cells were plated onto Luria-Bertani (LB) agar plates containing zeocin. A single colony was propagated overnight in LB broth, and plasmid DNA purified using a Qiagen miniprep kit. Restriction enzyme digests and electrophoresis were used to confirm the presence of the *nef* gene.

Bulk *nef* PCR products and clones were sequenced bi-directionally using the ABI Prism Big Dye Terminator v3.1 Cycle Sequencing Kit (Applied Biosystems). Sequence data were generated with the ABI 3130xl Genetic Analyzer (Applied Biosystems) and edited in Sequencher 4.8 (GeneCodes). *Nef* sequences were aligned to HXB2 and insertions with respect to HXB2 stripped out using HyPhy [62]. For each patient, CD4 and HLA-I downregulation was measured for a single *nef* clone with an intact open reading frame that was free of gross genetic defects, such as large deletions, and that was representative of the original bulk sequence by phylogenetic analysis [63]. HIV-1 subtypes of all *nef* clones were confirmed using the recombinant identification program (RIP; http://www.hiv.lanl.gov/content/sequence/RIP/RIP.html) and subtype grouping was corroborated in a maximum likelihood phylogenetic tree [63]. *Nef* clonal sequences are available as Genbank accession numbers KC906733-KC907077.

Subtype B and C consensus sequences (2004 consenses, available at http://www.hiv.lanl.gov/content/sequence/NEWALIGN/align.html) were commercially synthesized (Integrated DNA Technologies) and cloned into pSELECT-GFP. Site-directed mutagenesis of these templates was performed using the QuikChange XL system (Stratagene).

### CD4 and HLA-I downregulation assays

CD4 and HLA-I downregulation activity for each Nef clone was measured using a CEM-derived T cell line that expresses high levels of CD4 and HLA-A*02 (CEM-A*02). CEM T cells were transduced with a murine stem cell virus (MSCV) retroviral vector (Clontech) encoding human HLA-A*0201 and progeny were selected using puromycin. Surface expression of CD4 and HLA-A*02 was confirmed by flow cytometry, and double-positive cells were sorted. Cells used in experiments were maintained in R10 medium lacking puromycin for no longer than 3-4 weeks following puromycin selection. CEM-A*02 cells (300,000) were transfected by electroporation at 250 V and 950 μF with 4 μg of Nef clone and incubated for 20-24 hours. Separate CMV and human EF1 promoters in the pSELECT plasmid allow simultaneous expression of GFP and the *nef* insert, respectively, in transfected cells. Cells were stained at 20-24 hours post-transfection with APC-labeled anti-CD4 and PE-labeled anti-HLA-A*02 antibodies (BD Biosciences) and cell surface expression was measured in transfected (GFP-positive) cells by flow cytometry. For patient-derived Nef clones, the median fluorescence intensity (MFI) of CD4 or HLA-I expression in GFP-positive cells was normalized to the MFI of CD4 or HLA-I expression for the negative control (empty pSELECT-GFP plasmid) and positive control (SF2 *nef* cloned into the pSELECT-GFP plasmid) to determine the relative CD4 or HLA-I downregulation capacity: (negative control – patient Nef)/(negative control – positive control). A normalized MFI value of 0% indicates no downregulation activity and a value of 100% indicates downregulation capacity equivalent to that of the positive control, respectively. For a subset of Nef clones (n = 24, representing a range of HLA-A*02 downregulation functions), HLA-B downregulation activity was measured using a CEM cell line stably expressing HLA-B*07 (generated similarly using MSCV) and a PE-labeled anti-HLA-B*07 antibody (BD Biosciences). All assays were performed in duplicate and results are presented as the mean of these measurements.

### Western blot analysis

Steady state Nef protein levels were measured by Western blotting for all Nef clones that displayed poor (<35%) function for both CD4 and HLA-I downregulation (n = 22 total; 4 subtype A, 2 subtype B, 14 subtype C and 2 subtype D) as well as a random selection of 60 clones with CD4 and HLA-I downregulation functions above this threshold. Positive (SF2 Nef) and negative (empty pSELECT vector) controls were included in all Western blot experiments. A total of 1 × 10^6^ CEM cells were transfected by electroporation with 10 μg of Nef clones. After 24 hours, cell pellets were collected for preparation of total cell lysates as described previously [57]. Samples were subjected to SDS-PAGE and proteins electro-blotted onto nitrocellulose membrane. HIV-1 Nef was detected using rabbit polyclonal anti-HIV-1 Nef serum (1:5,000 dilution) (NIH AIDS Research and Reference Reagent Program, USA) primary antibody, followed by horseradish peroxidase (HRP)-conjugated donkey anti-rabbit IgG (1:50,000) (Amersham Biosciences). Nef clones that exhibited poor detection were subsequently probed using sheep polyclonal anti-HIV-1 Nef serum ARP 444 (1:2,000) (NIBSC Center for AIDS Reagents, UK) primary antibody, followed by HRP donkey anti-sheep IgG (1:50,000) (Jackson ImmunoResearch Europe Ltd). Actin expression was simultaneously quantified in all experiments. Band intensities were quantified using ImageQuant LAS 4000 (GE Healthcare Life Sciences).

### Statistical analysis

Nef CD4 and HLA-I downregulation capacities were compared between subtypes using Kruskal-Wallis with Dunn’s multiple comparisons post-hoc tests. Spearman’s or Pearson’s correlation were used to assess the relationships between Nef-mediated activities and between Nef CD4/HLA-I downregulation capacities and Nef sequence length. Socio-demographic and clinical parameters were compared among subtypes using the Kruskal-Wallis test for continuous variables and Fisher’s Exact Test for categorical variables.

The relationship between Nef function and socio-demographic or clinical factors was assessed by calculating univariate and multivariable linear regression. Variables investigated were sex (male vs. female [reference group]), age (per year increment), log_10_ plasma viral load (per log_10_ increment), logCD4 count (per log_10_ increment) and HIV-1 subtype (reference group subtype B). All variables p < 0.1 in the univariate analysis were included in the multivariable model.

In analyses stratified by subtype, the Mann-Whitney U test was used to identify common amino acid variants associated with significantly increased or decreased Nef-mediated CD4/HLA-I downregulation function. A frequency cut-off (n ≥ 5) was used to exclude rare amino acid variants. Q-values, the p-value analogue of the false discovery rate (FDR), were calculated to account for multiple comparisons [64]. Fisher’s exact test, with Bonferroni correction for multiple tests, was used to test for significance of differences >25% in the frequency of amino acids between subtype C sequences with high function and those with the lowest function. Inter-subtype differences in the frequencies of specific amino acids were investigated using the Chi-square test [45]. The significance cut-off for all analyses was p < 0.05, and q ≤ 0.4 where appropriate.

## Competing interests

The authors declare that they have no competing interests.

## Authors’ contributions

MAB, ZLB and TN designed the study; JKM, HB, XTK, AQL, PM, SO, GQL, BB, and RD, performed the experiments and/or collected data; JKM, CJB, EM, RMM, and ZLB developed analytical methods and/or analyzed data, CM, DRB, PWH, PJRG, BDW, PRH, JNM. and TN provided access to patient samples and cohort data; and JKM, MAB and ZLB, wrote the paper. All authors read and approved the final manuscript.

## Supplementary Material

Additional file 1: Table S1 Created using Microsoft Excel. Contains HXB2-aligned, gap-stripped Nef amino acid sequences from all clones and their associated CD4 and HLA-I downregulation functions. Click here for file
